# Atomic Point Contact Raman Spectroscopy of a Si(111)-7 × 7 Surface

**DOI:** 10.1021/acs.nanolett.1c00998

**Published:** 2021-05-02

**Authors:** Shuyi Liu, Adnan Hammud, Martin Wolf, Takashi Kumagai

**Affiliations:** † Department of Physical Chemistry, Fritz-Haber Institute of the Max-Planck Society, Faradayweg 4-6, Berlin 14195, Germany; ‡ Department of Inorganic Chemistry, Fritz-Haber Institute of the Max-Planck Society, Faradayweg 4-6, Berlin 14195, Germany; § Center for Mesoscopic Sciences, Institute for Molecular Science, Okazaki 444-8585, Japan

**Keywords:** tip-enhanced Raman spectroscopy, low-temperature scanning tunneling microscopy, Si(111)-7 × 7 surface, atomic point contact

## Abstract

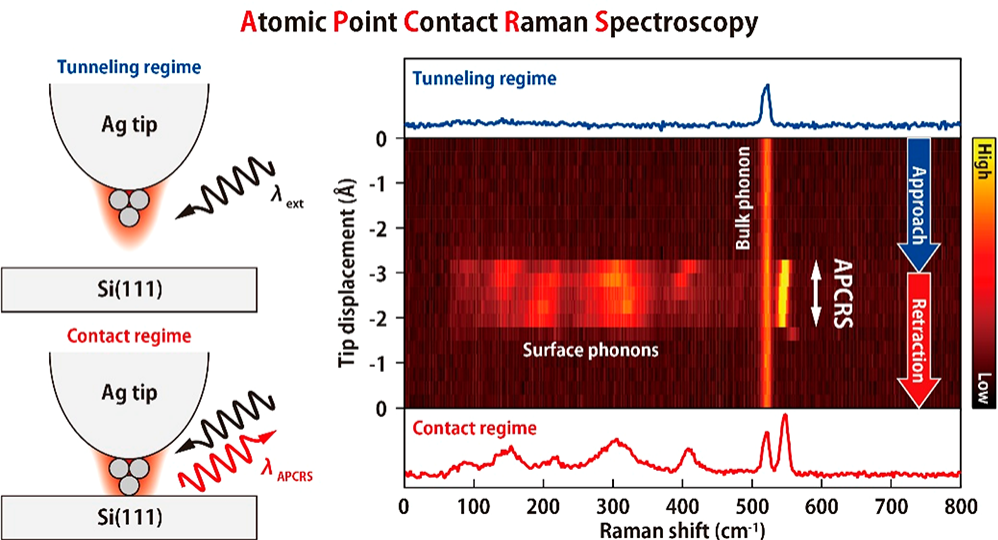

Tip-enhanced Raman scattering (TERS) has recently demonstrated the exceptional sensitivity to observe vibrational structures on the atomic scale. However, it strongly relies on electromagnetic enhancement in plasmonic nanogaps. Here, we demonstrate that atomic point contact (APC) formation between a plasmonic tip and the surface of a bulk Si sample can lead to a dramatic enhancement of Raman scattering and consequently the phonons of the reconstructed Si(111)-7 × 7 surface can be detected. Furthermore, we demonstrate the chemical sensitivity of APC-TERS by probing local vibrations resulting from Si–O bonds on the partially oxidized Si(111)-7 × 7 surface. This approach will expand the applicability of ultrasensitive TERS, exceeding the previous measurement strategies that exploit intense gap-mode plasmons, typically requiring a plasmonic substrate.

Plasmonic nanostructures can be used to manipulate electromagnetic fields well below the diffraction limit and to largely enhance light–matter interactions, offering manifold applications in nanoscale science and technology.^1^ The plasmonic field confinement at the scale of tens of nanometers is readily achieved with various nanostructures.^2^ Even more tightly confined fields can be generated in ultrathin dielectric gaps (<1 nm) between metals,^3^ although the precise and reproducible control of such tiny gaps is a challenging task. The extreme confinement of plasmonic fields has attracted increasing attention in nanophotonics due to potential applications in nonlinear nanooptics, cavity optomechanics, quantum optoelectronics, ultrasensitive and high-spatial-resolution optical spectroscopy.^4^ In particular, the crucial role of atomic-scale structures in the field confinement and enhancement has been highlighted in a plasmonic “picocavity”, where the ångström-scale field confinement occurs at atomic-scale protrusions.^5−7^ However, in experiments, the stability of atomic-scale protrusions is a key issue to exploit atomistic plasmonic fields.^8^ Cooling the system to cryogenic temperature provides exceptional thermal stability. Remarkably, low-temperature tip-enhanced Raman spectroscopy (TERS) has recently proved the unprecedented chemical sensitivity with even submolecular spatial resolution.^9−11^ TERS is a promising technique in wide-ranging fields including single-molecule spectroscopy,^9−11^ electrochemistry,^12,13^ heterogeneous catalysis,^14,15^ biomolecular identification,^16−18^ and 2D materials characterization,^19−23^ bearing a great potential as nanoscale chemical microscopy. In most cases, however, the underlying enhancement mechanism of the Raman scattering intensity relies largely on the extreme field enhancement in plasmonic nanogaps.^24−27^ This imposes a severe limitation on measurable systems, thus typically requiring a plasmonic substrate. As an attempt to overcome this obstacle, here we demonstrate that an atomic point contact (APC) between a plasmonic tip and a nonplasmonic surface can dramatically enhance Raman scattering and that the phonon modes of the Si(111)-7 × 7 surface can be observed.

Figure 1a depicts the schematic of the experiment. The STM junction consisting of a Ag tip and the reconstructed Si(111)-7 × 7 surface at 10 K is illuminated by a 633(532) nm narrow-band laser, generating a strong plasmonic field localized on the tip. Typically, the tip has an apex diameter of several tens of nm which couples the propagating electromagnetic field through localized surface plasmon excitation. Additional confinement will occur at the atomic-scale protrusion (single atoms) existing at the end of the tip, yielding the extreme field confinement and enhancement through the atomic-scale lightning effect.^6,7,28^ The Raman signal from the junction is collected in the backscattering geometry. Figure 1b shows a scanning tunneling microscopy (STM) image of the empty states of the Si(111)-7 × 7 surface scanned under illumination. We found that the illumination during scanning creates atomic defects in the topmost layer, which appear as a pair of bright and dark spots (Figure 1c). These defects occur only in faulted half unit cells (FHUCs) of the reconstructed Si(111)-7 × 7 surface.^29^ The dark/bright pair is attributed to the removal and subsequent rebinding of a Si adatom. A similar illumination-induced defect formation was reported previously.^30,31^ The Si(111)-7 × 7 surface exhibits a surface state near the Fermi level and undergoes a transition from metallic to insulating behavior below ∼20 K.^32^ The surface state under illumination is confirmed by scanning tunneling spectroscopy (STS) as shown in Figure 1d, and similar spectra were obtained under illumination of 633 nm. Although the appearance of the surface state is consistent with observations for the nonilluminated surface, its energy is down-shifted by ∼0.5 eV in the STS under illumination.^32^ This suggests that the surface is positively charged, causing band bending at the surface.^33^ As the FHUCs have a larger local density of states near the Fermi level than that of unfaulted half unit cells (UHUCs),^32^ the illumination may result in more charging in FHUCs, which could promote the bond rupture of the Si adatoms.

**Figure 1:**
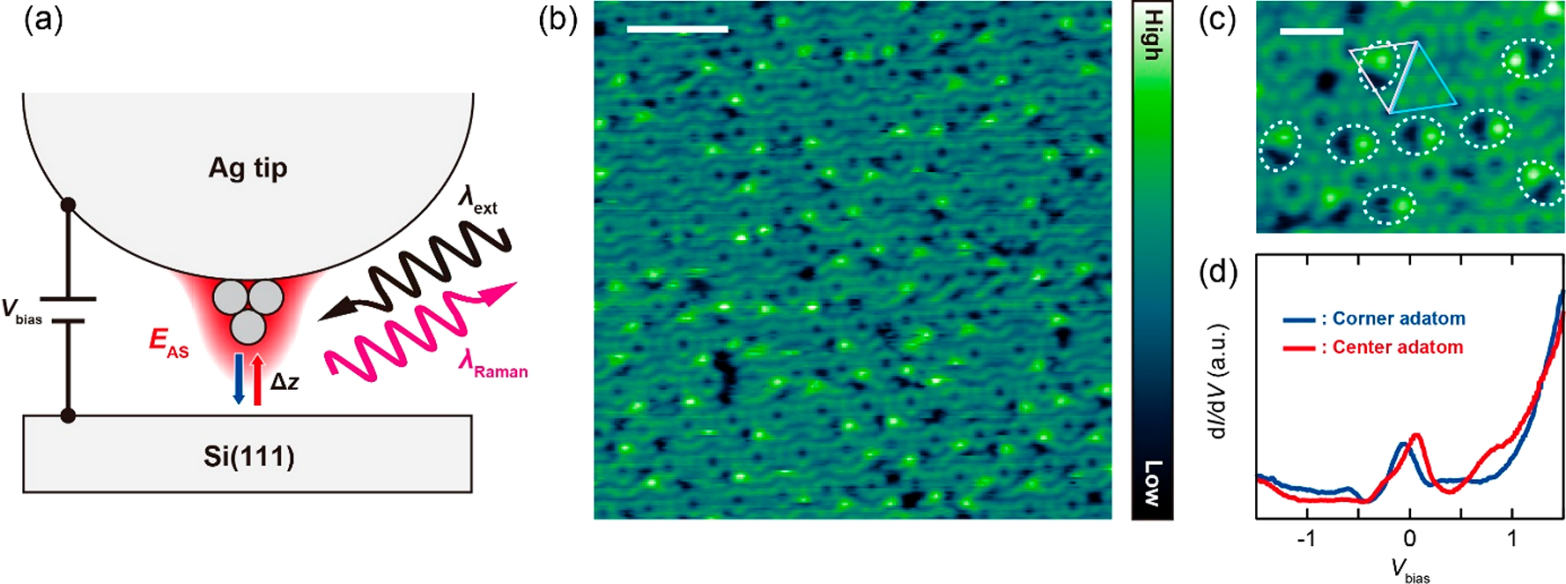
(a) Schematic of the experiment. *E*_AS_ illustrates the extreme plasmonic field occurring at the atomic-scale protrusion at the tip apex. (b) STM image of Si(111)-7 × 7 under illumination (10 K, *V*_bias_ = 0.3 V, *I*_STM_ = 1 nA, λ_ext_ = 633 nm, *P*_ext_ = 0.7 W/cm^2^, scale bar = 5 nm). (c) Enlarged STM image (scale bar = 1 nm). The bright/dark pairs are indicated by the dashed ellipses. The faulted/unfaulted half unit cell is indicated by the pink/blue triangle. (d) Conductance spectrum measured under illumination (10 K, set-point: *V*_bias_ = 0.5 V, *I*_STM_ = 0.1 nA, λ_ext_ = 532 nm, *P*_ext_ = 0.15 mW/μm^2^).

Figure 2a shows a waterfall plot of the TERS spectra recorded while vertically approaching and retracting the tip to and from a corner adatom in an UHUC. The vertical and horizontal axes correspond to the tip–surface distance (Δ*z*) and the Raman shift, respectively, and the color scale represents the Raman intensity. At large Δ*z* only the optical phonon mode of the bulk Si is observed at 520 cm^–1^ (top panel of Figure 2a).^34^ No characteristic TERS signals appear as the Δ*z* decreases in the tunneling regime, however, a sudden, dramatic change takes place when the tip contacts the surface, whereas the intensity of the bulk mode is not affected. As can be seen in the current–distance (*I*_STM_–Δ*z*) curve in the left panel of Figure 2a, the *I*_STM_ increases (decreases) monotonically in the tunneling regime, but it saturates when the contact between the tip apex and the surface is formed. This is a typical behavior observed for the APC formation.^35^ In the following, we refer to the TERS measurement in the contact regime as APC-TERS.

**Figure 2:**
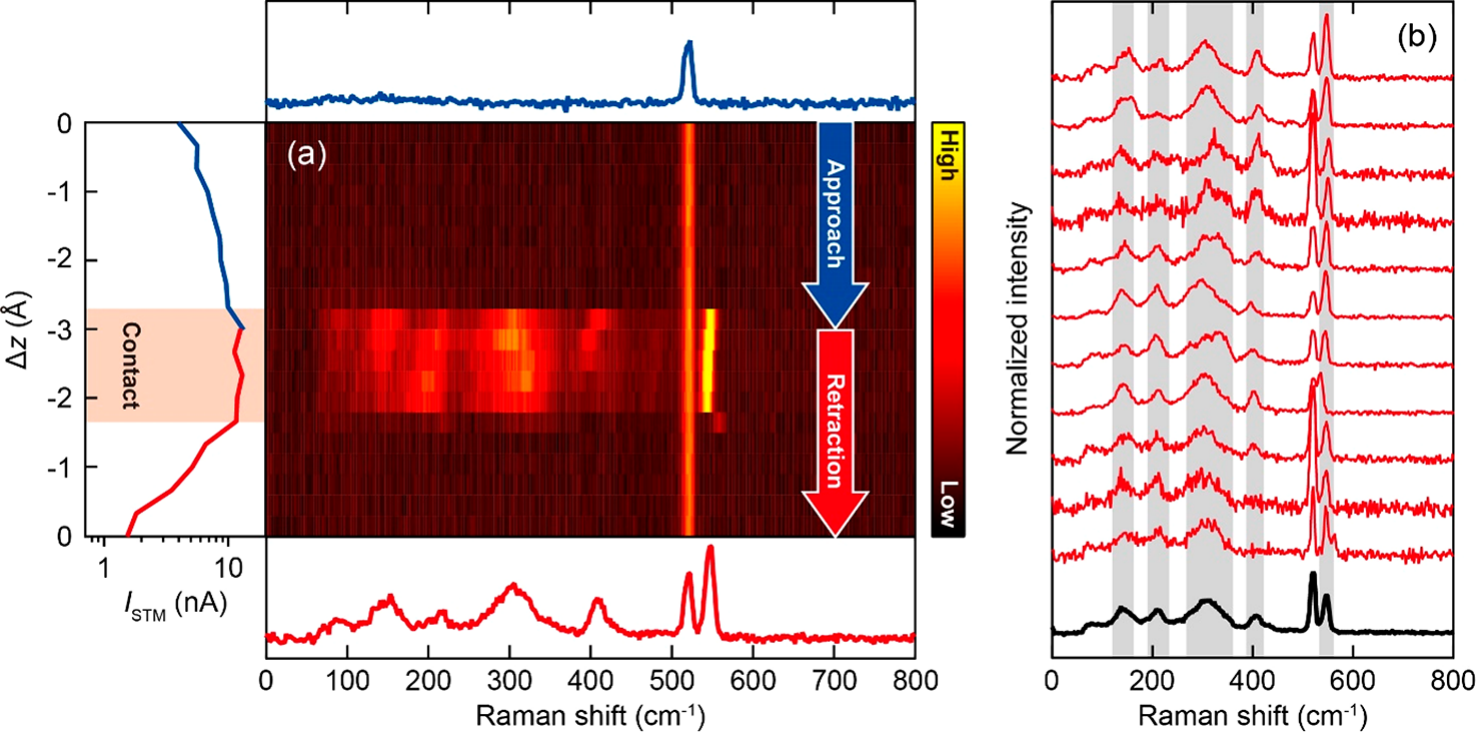
(a) Water fall plot of TERS recorded during tip-approach and retraction over Si(111)-7 × 7 (10 K, *V*_bias_ = 0 V, λ_ext_ = 633 nm, *P*_ext_ = 0.7 mW/μm^2^). The left panel shows the simultaneously recorded *I*_STM_–Δ*z* curve. Although the *V*_bias_ is nominally set to zero, the current occurs due to the photovoltage under illumination. The red shaded region indicates the APC. The top and bottom panels display the TERS spectra in the tunneling and contact regimes, respectively. (b) TERS spectra obtained for 11 different APCs (red) recorded for different location (UHUCs) and different tip conditions. The black one shows the averaged spectrum. The spectra are normalized to its area from range of Raman shift 0–500 cm^–1^.

The onset of the saturation of the *I*_STM_ coincides with the dramatic changes in TERS. In the APC regime, characteristic Raman peaks appear at 100–450 cm^–1^ and at 550 cm^–1^ which are assigned to surface phonons of the Si(111)-7 × 7 surface (as discussed below). The strong TERS signals disappear when the tip is retracted and the tip–Si bond is broken. It should be noted that, after APC-TERS measurement and retraction of the tip, a small Ag cluster always appeared on the surface (Figure S1), indicating a bond formation between the tip apex atoms and the Si surface. This causes the asymmetric behavior of the TER spectra and the *I*_STM_–Δ*z* curve with respect to the turning point of the sequential approach and retraction (Δ*z* = −3 Å) as seen in Figure 2a. Although the exact peak positions and the intensities of APC-TERS depend on the tip conditions, the dramatic enhancement is highly reproducible (Figure S2). Furthermore, spectral shifts of the peaks occur as the tip is further squeezed into the surface (as well as during retraction), which potentially result from mechanical stress and/or charge transfer^36^ accompanied by structural deformation of the junction.

To evaluate the APC-TERS spectra of the Si(111)-7 × 7 surface, we selected the spectrum at the moment of the APC formation from repeated measurements because at this point the surface structure should not be disturbed significantly. The reproducibility of these spectra is confirmed by repeating the measurement under different tip conditions (Figure 2b). A small difference in the peak positions can be attributed to slightly different bonding geometries of the APC. We also confirmed that an Au tip gives the similar results (Figure S3), corroborating that the observed peaks in APC-TERS can be assigned to the vibrations of the Si surface. In the averaged spectrum, peaks at 150, 210, 310, 400, and 550 cm^–1^ are resolved, which are in good agreement with the simulated surface phonon modes of Si(111)-7 × 7.^37^ The observed Raman peaks originate from the phonons that extend over a substantial momentum space in the Brilliouin zone due to spatial localization of the confined field in the APC, leading to a large momentum uncertainty. Therefore, the surface phonons observed in APC-TERS appear broader than the bulk phonon at 520 cm^–1^. A similar mechanism has been proposed for near-field optical excitation of Si.^38,39^ In addition, the peaks between 150 and 380 cm^–1^ might be further broadened by coupling to the Ag phonons of the tip, which serves as an additional damping channel.^40^

The enhancement of TERS is generally attributed to two mechanisms, namely electromagnetic (EM)^41^ and charge-transfer (chemical)^42^ effects. The APC formation results in hybridization between the Ag atom at the tip apex and the surface Si atom(s), which modifies the electronic structure. This may significantly affect the Raman scattering cross section through the charge-transfer mechanism.^43,44^ As discussed in our previous publication,^45^ the reactivity of the surface, and thus the degree of the hybridization, has a decisive impact on the enhancement caused by the ACP formation. The important role of surface reactivity is further corroborated by examining ACP-TERS for a highly oriented pyrolytic graphite (HOPG), a very inert surface, which does not show dramatic enhancement upon a contact formation, as weak hybridization will not lead to changes in the electronic structure. The EM enhancement mechanism is also operative as the intensity of APC-TERS is correlated with the plasmon response of the tip (Figure S4). In addition to these two general mechanisms, the current-driven Raman scattering process in TERS was also discussed recently,^46^ which should also play a role for APC-TERS. Additionally, light–matter coupling should be further enhanced in the vicinity of the APC because of extreme (atomic scale) field confinement. We find that the Raman enhancement is significantly reduced as the tip is further squeezed into the surface by ∼2 Å from the onset of APC formation (Figure S5). This implies attenuation of the atomic-scale field caused by a change of the APC configuration (probably breakdown of the single APC).

APC-TERS measured over different Si adatoms do not exhibit significant differences in either FHUCs or UHUCs. Most probably their subtle spectral differences are overwhelmed by different tip conditions (slightly different geometries of the APC). However, clear spectral changes can be observed when APC-TERS is recorded at step edges (Figure 3a and Figure S6) where the peak positions are clearly shifted compared to those over the flat surface. Especially, more peaks appear around ∼400 cm^–1^, where the collective mode of the surface vibrations is located.^47^ In contrast to the other modes that are more localized at the adatom (at 150, 210, 310 cm^–1^), the collective mode involves the substantial motion of the atoms in the underneath layers.^37^ This mode could be split at the step edge because of the symmetry reduction (lateral periodicity of the surface perpendicular to the step direction is broken), leading to multiple peaks.

**Figure 3:**
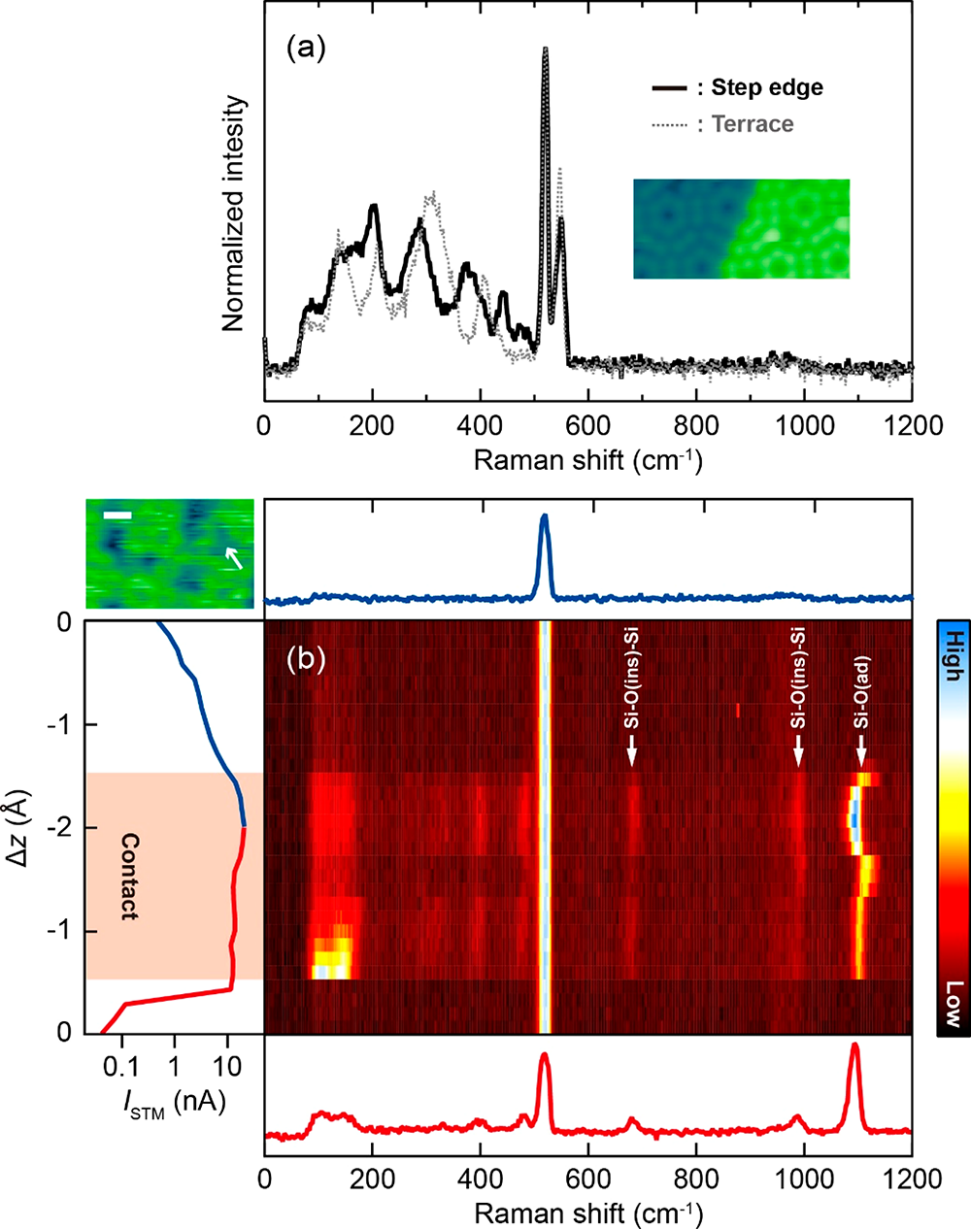
(a) APC-TERS at the step edge of the Si(111)-7 × 7 surface (10 K, *V*_bias_ = 0 V, λ_ext_ = 633 nm, *P*_ext_ = 0.7 mW/um^2^). The gray dashed line is the averaged spectrum over the terrace. The inset shows the STM image of the step edge. (b) APC-TERS at oxidized region of the Si(111)-7 × 7 surface (10 K, *V*_bias_ = 0 V, λ_ext_ = 532 nm, *P*_ext_ = 1 mW/μm^2^). The left panel shows the simultaneously recorded *I*_STM_–Δ*z* curve. The red shaded region indicates the APC. The top and bottom panels display the TERS spectra in the tunneling and contact regimes, respectively. The STM image of the partially oxidized surface is shown in top-left (scale bar is 2 nm). The position for APC-TERS measurement is indicated by the arrow.

To test the chemical sensitivity of APC-TERS, we also investigated the partially oxidized Si(111)-7 × 7 surface. The dark areas in the inset STM image (top-left) of Figure 3b correspond to the surface oxide.^48^ When APC-TERS is recorded at this dark area, three peaks are observed at higher frequency (>650 cm^–1^) where no Raman signals occur from the bare Si(111)-7 × 7 surface. The peak at 683 cm^–1^ involves the inserted O atom, denoting as O(ins), moving perpendicular to the Si–O(ins)–Si bond and the peak at 989 cm^–1^ results from the O atom moving along this bond.^49,50^ The peak at 1108 cm^–1^ may be assigned to the stretching of Si–O(ad) in which the O atom is chemically adsorbed on the adatom. This mode is red-shifted in APC-TERS compared to other experiments,^50−52^ possibly because the bond is softened by the contact with the Ag tip (causing charge transfer from the Si surface). We found that these spectral signatures are characteristic for the dark (oxidized) areas and that multiple peaks are observed at the range of stretching of Si–O(ins)–Si (see also Figure S7), indicating further oxidized products. Because of the destructive nature of the APC (Figure S1), Raman imaging was impossible. However, the above results demonstrate that APC-TERS will be capable of studying local chemical structures on the atomic scale by combing with STM images. It may be possible to record APC-TERS mapping for a moderately reactive tip/surface and a controlled modification of the tip apex, for example, attaching a single molecule, would be a potential approach.

APC-TERS is, in principle, applicable to nonplasmonic substrates and the exceptional sensitivity will be obtained for many other materials. The strong TERS signal only appeared for the APC configuration, suggesting that atomic-scale structures are indispensable to understand light–matter interactions in metal–semiconductor hybrid nanosystems.

## Supporting Information Available

Methods, small Ag clusters created after APC-TERS measurement, TERS for different APCs over Si(111)-7 × 7, APC-TERS of Si(111)-7 × 7 measured with an Au tip, LSPR dependence of APC-TERS, wavelength dependence of APC-TERS and attenuation of the intensity in the contact regime, APC-TERS at step edges of Si(111)-7 × 7, APC-TERS over partially oxidized Si(111)-7 × 7 (PDF)
