# Supplementary material for: Atomic Point Contact Raman Spectroscopy of a Si(111)-7 × 7 Surface
Source: Nano Lett. 2021 May 2;21(9):4057–61. doi: 10.1021/acs.nanolett.1c00998 (PMC8288640; doi:10.1021/acs.nanolett.1c00998)
Supplement: Supplementary file 1 — nl1c00998_si_001.pdf [file nl1c00998_si_001.pdf]

Supporting Information:

Atomic Point Contact Raman Spectroscopy of a Si(111)-7×7 Surface

Shuyi Liu[1], Adnan Hammud[2], Martin Wolf[1], Takashi Kumagai[1,3]*

[1]*Department of Physical Chemistry, Fritz-Haber Institute of the Max-Planck Society, Faradayweg 4-6, 14195 Berlin, Germany*

[2]*Department of Inorganic Chemistry, Fritz-Haber Institute of the Max-Planck Society, Faradayweg 4-6, 14195 Berlin, Germany.*

[3]*Center for Mesoscopic Sciences, Institute for Molecular Science, Okazaki 444-8585, Japan*

*Corresponding author: kuma@ims.ac.jp

Table of Contents

1. **Methods**
2. **Small Ag clusters created after APC-TERS measurement**
3. **TERS for different APCs over Si(111)-7×7**
4. **APC-TERS of Si(111)-7×7 measured with an Au tip**
5. **LSPR dependence of APC-TERS**
6. **Wavelength dependence of APC-TERS and attenuation of the intensity in the contact regime**
7. **APC-TERS at step edges of Si(111)-7×7**
8. **APC-TERS over partially oxidized Si(111)-7×7**

1. Methods

Sample preparation. All experiments were performed in ultra-high vacuum (UHV) chambers (base pressure <5×10^{-10} mbar). We used an n-doped Si(111) wafer (resistance: 0.03–0.08 Ω, thickness: 0.5 mm). The sample was degassed at ~800 K in the chamber overnight, then it was annealed several times by direct current heating to ~1470 K for 10 seconds. For partial oxidation of the Si(111)-7×7 surface, the clean surface was exposed to an oxygen gas (2.4 Langmuir) at room temperature.

STM measurement. We used a low-temperature STM from UNISOKU Ltd. (modified USM-1400) operated with Nanonis SPM Controller (SPECS GmbH). The bias voltage (V_{bias}) was applied to the sample, and the tip was grounded. The tunneling current (I_t) was collected from the tip. We used Ag tips fabricated by focused ion beam milling, see details in Ref. 1.

TERS measurement. The excitation laser was focused to the STM junction with an *in-situ* Ag-coated parabolic mirror (numerical aperture of ~0.6) mounted on the cold STM stage. The parabolic mirror was precisely aligned using piezo motors (Attocube GmbH), which allow three translational and two rotational motions. For the Raman measurements we used a HeNe laser for 633 nm and a solid-state laser for 532 nm (Cobolt). The incident beam is linearly polarized along the tip axis (p-polarization). The scattered photons are collected by the same parabolic mirror and detected outside of the UHV chamber with a grating spectrometer (AndorShamrock 303i).

2. Small Ag clusters created after APC-TERS measurement

Figure S1 shows a typical STM image of the Si(111)-7×7 surface after APC-TERS measurements. We recorded APC-TERS twice in this area and the Ag clusters were formed on the surface, which were dropped off from the tip apex.

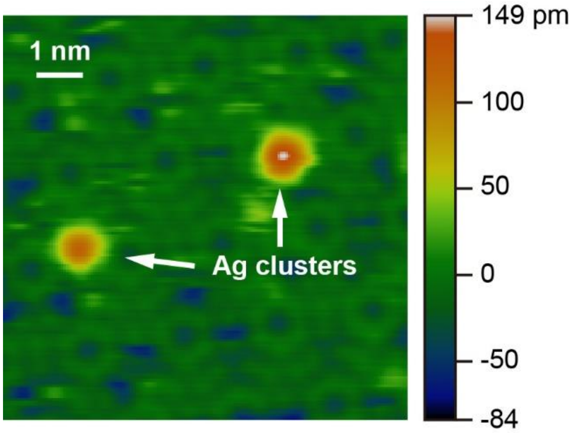

Figure S1. STM image of the Si(111)-7×7 after APC-TERS measurement (10 K, V_{bias}=0.5 V, I_{STM}=30 pA).

3. TERS for different APCs over Si(111)-7×7

The dramatic enhancement of Raman scattering at APCs is reproducible for different tips. **Figure S2** shows the waterfall plots of the APC-TER spectra recorded under two different tip conditions by approaching and retracting the tip to and from the Si surface. As described in main text, there is no TERS signal in the tunneling regime but it appeared when the Ag tip contacts with the surface. Spectral diffusion in the contact regime (when the tip is further squeezed into the surface) shows a tip-condition dependence. However, the peak positions at the moment of the APC formation are very similar.

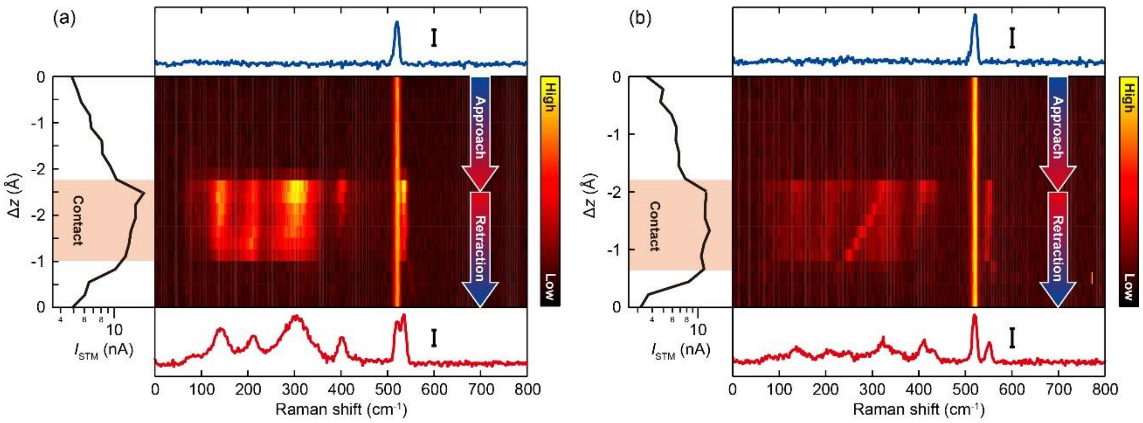

Figure S2. (a–b) Water fall plot of TERS recorded during tip-approach and retraction over Si(111)-7×7 (10 K, V_{bias}=0 V, λ_{ext}=633 nm, P_{ext}=0.7 mW/μm^2). The left panel shows the simultaneously recorded I_{STM}–Δz curve. The orange shaded region indicates the APC. The top and bottom panels display the TER spectra in the tunneling and contact regimes, respectively.

4. APC-TERS of Si(111)-7×7 measured with an Au tip

Figure S3 (bottom) shows the APC-TER spectrum recorded by an Au tip with illumination at 633 nm. The peak positions are consistent with the case recorded by Ag tips (top).

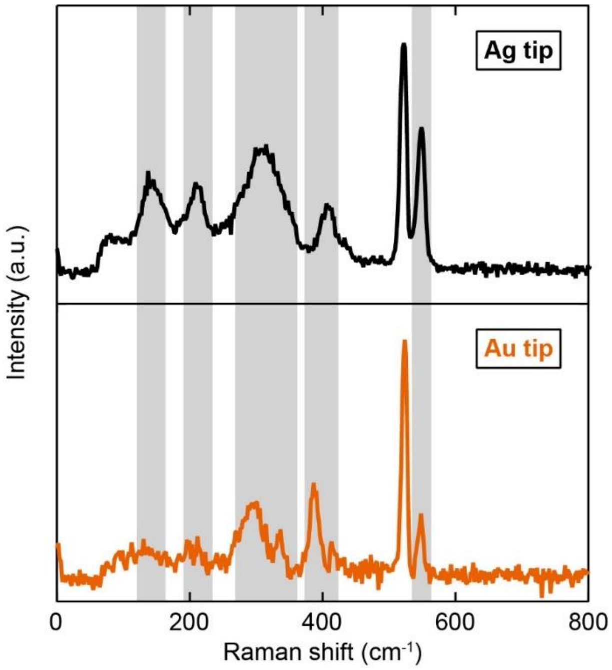

Figure S3. APC-TERS of the Si(111)-7×7 surface measured with an Au tip (10 K, V_{bias}=0 V, λ_{ext}=633 nm, P_{ext}=0.33 mW/μm^2).

5. LSPR dependence of APC-TERS

The absolute intensity of APC-TERS is correlated with the plasmon response of the tip. **Figure S4a** shows Scanning tunneling luminescence (STL) spectra recorded over the Si(111)-7×7 surface under four different tip conditions. The junction is illuminated with wavelength at 633 nm during the measuring in order to make the Si surface conductive. The sharp peaks at 654 nm corresponds to Raman scattering of the bulk Si (optical phonon mode). The continuous background is the current-induced luminescence. As shown in **Fig. S4b**–**S4e**, the APC-TERS intensity is larger for the tips exhibiting a stronger plasmon response (more intense STL intensity).

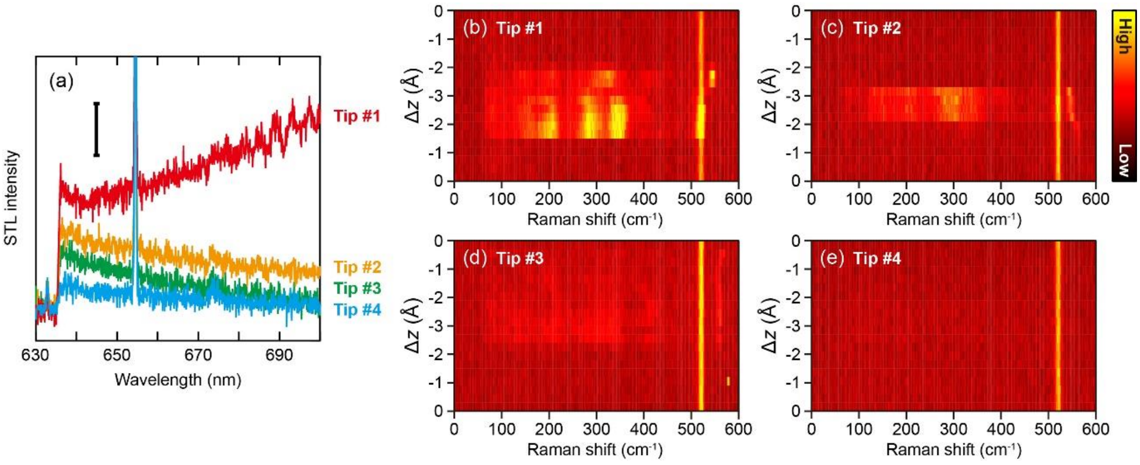

Figure S4. (a) STL spectra recorded over Si(111)-7×7 under illumination for different tip conditions (10 K, V_{bias}=3 V, I_{STM} = 30 nA, λ_{ext}=633 nm, P_{ext}=0.7 mW/μm^2). (b–e) Waterfall plots of TERS recorded for respective tips in (a) (10 K, V_{bias}=0 V, λ_{ext}=633 nm, P_{ext}=0.7 mW/μm^2).

6. Wavelength dependence of APC-TERS and attenuation of the intensity in the contact regime

Figure S5 shows a waterfall plot of the APC-TER spectra recorded with illumination at 532 nm. The Ag tip is deeply moved into the Si surface. The dramatic TERS enhancement only happens within the regime of ~2 Å from the ACP formation. However, the intensity is significantly attenuated as the tip is further squeezed into the surface.

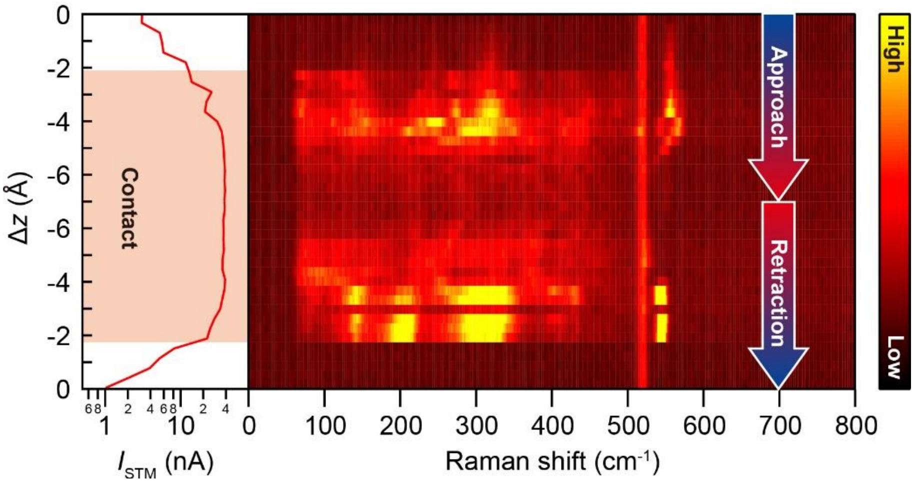

Figure S5. Water fall plot of TERS recorded during tip-approach and retraction over the Si(111)-7×7 surface (10 K, V_{bias}=0 V, λ_{ext}=532 nm, P_{ext}=0.7 mW/μm^2). The left panel shows the simultaneously recorded I_{STM}–Δz curve.

7. APC-TERS at step edges of Si(111)-7×7

Figure S6 shows TER spectra at the moment of ACP formation at the step edges of the Si(111)-7×7 surface measured under different tip conditions. Although the intensity depends on the tip conditions (see **Fig. S4**), the peak positions are similar in all the measurements.

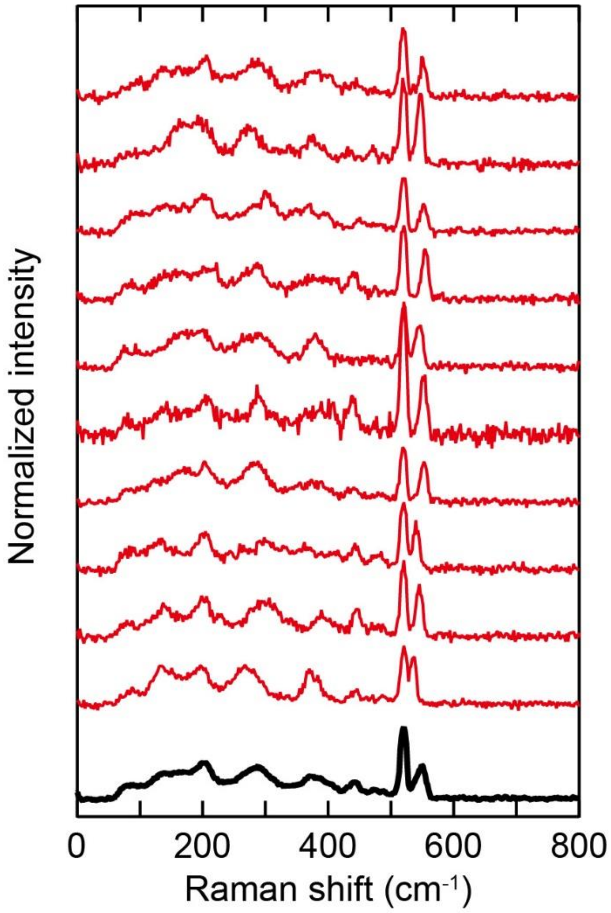

Figure S6. APC-TER spectra obtained for 10 different APCs at the step edges (red) and the averaged one (black). The spectra are normalized by the averaged intensity in the range of 0–500 cm^{-1}.

8. APC-TERS over partially oxidized Si(111)-7×7

As discussed in the main text, we found that the APC-TER spectra over a partially oxidized Si(111)-7×7 show a strong position dependence. **Figure S7** displays the APC-TER spectra obtained at different locations.

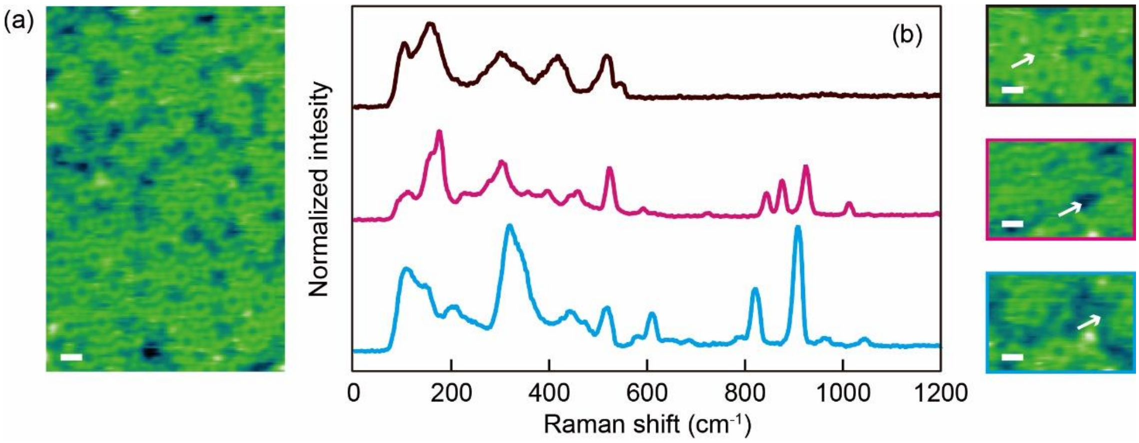

Figure S7. (a) STM image of the partially oxidized Si(111)-7×7 surface. (b) APC-TER spectra recorded over the clean area (black) and the oxidized part (red and blue). The measurement position is indicated in the STM images in the right.

References

[1] Böckmann, H.; Liu, S.; Müller, M.; Hammud, A.; Wolf, M.; Kumagai, T. Near-Field Manipulation in a Scanning Tunneling Microscope Junction with Plasmonic Fabry-Pérot Tips. *Nano Lett.* **2019**, *19* (6), 3597–3602.
